# Mechanical vestibular stimulation versus traditional balance exercises in children with Down syndrome

**DOI:** 10.4314/ahs.v22i1.46

**Published:** 2022-03

**Authors:** Ibrahim M Nahla, Salem Elham El-Sayed, Abd-Elraouf Ehab Ragaa, Abd El hamid Amr Abd El Ghafar

**Affiliations:** 1 Faculty of Physical Therapy, Cairo University, Giza, Egypt; 2 Faculty of Medicine, Cairo University, Giza, Egypt

**Keywords:** Balance exercises, down syndrome, mechanical vestibular stimulation

## Abstract

**Background:**

regaining balance control is the key to decrease risk of falls in children with Down syndrome.

**Objectives:**

To compare between the effect of mechanical vestibular stimulation and balance exercises on balance in children with Down syndrome.

**Methods:**

Thirty children participated in the study. They were divided randomly and equally into; group A and group B, both groups received the designed program with regular balance exercises for group A and mechanical vestibular stimulation for group B, treatment was conducted for one hour 3 times per week for 3 successive months. Balance as stability indexes (regarding anteroposterior, mediolateral and over all stability indexes) was evaluated before and after treatment by Biodex balance system.

**Results:**

T-test was conducted to compare the mean values of stability indexes between groups. Non-significant difference between groups was recorded before treatment (p value > 0.05), while improvement was recorded when comparing post and pretreatment results for both groups (p > 0.0001). More significant improvement was recorded for group B when comparing the post treatment results with group A (p > 0.05).

**Conclusion:**

Mechanical vestibular stimulation is better added to the rehabilitation program to improve balance in children with Down syndrome.

## Introduction

Children with Down syndrome usually experience difficulty in maintaining balance due to one or more of the following causes as hypotonia, laxity of ligaments, poor postural control and muscle weakness, this is beside the below normal average intelligence, that results in delay in the acquisition of motor skills^1^. Although, generally the gross motor skills are consistently low compared to those of normal children but, lack of adequate balance control is still the hardest function to acquire which affects their abilities to accomplish independent skills and restricts their quality of performance for the activities of daily living^2^.

Balance is the ability to maintain the body in equilibrium or to keep the center of mass into the base of support to maintain a stable stance position and prevent falls. When balance is disturbed regaining position can either by movement of one or more of body segments to relocate the center of mass or adjust the width of the base of support by taking a step^3^. Years ago, the Biodex Balance System (BBS) was used to evaluate postural stability and balance^4^ as it measures the individual abilities to stabilize his position when exposing to dynamic disturbance by recording the antero-posterior and medio-lateral deviations simultaneously.

Balance exercises had been used to improve the abilities of children with Down syndrome to control their postural stabilities, as exercises on tilting board using different positions (quadruped, kneeling, half kneeling and standing) and facilitation of postural reactions in forward, backward and sideways.

Afferent information for postural control system comes from visual, vestibular and somatosensory inputs. Visual inputs that collect information concerning the position and motion of the head with respect to the surrounding objects. The vestibular system provides the Central Nervous System (CNS) with information about the position and movement of the head in relation to gravity, while the somatosensory inputs give information about the orientation of body parts to each other and to the supporting surface^7^.

The purpose of therapeutic vestibular stimulation is to increases the brain's capabilities to organize different stimuli to correct motor response that help children with impairment of sensory motor integration to achieve motor activities to help them actively stimulate their vestibular system. For relaxation, slow and regular stimulation is used for spastic children, but fast and irregular rhythm can be used for hypotonic children to help in increasing the attention and arousal levels due to increase of the vestibular stimulation^8^.

Mechanical vestibular stimulation help readjustment of postural control through integration of external visual, auditory and vestibular feedback that is believed to be an effective therapy for improving balance control. It is thought that by giving additional vestibular information, they will become more aware of the body's center of mass displacements and orientation in space^9^. Rotating or accelerating spinning movement, results in stimulation of the labyrinth receptors which produce the vestibulo-ocular reflex (VOR) that causes stimulation of the semicircular canals to mechanically integrate the different sensed inputs and send feedback information to the brain about body position.

So, the aim of the study was to compare between the effect of mechanical vestibular stimulation and the traditional balance exercises on balance in children with Down syndrome.

## Study design

### Randomized Controlled Trial

#### Subject

The purpose and procedure of the research were presented according to the guidelines of the Declaration of Helsinki. All parents gave their written consent for participation of their children in the examination and treatment program. The project of the research was approved by the ethical committee board of Faculty of Physical Therapy, Cairo university (P.T.REC/012/001795). Study was conducted at the outpatient clinic Faculty of Physical Therapy, Cairo University during the period from December 2019 to March 2020.

Thirty-five children diagnosed as Down syndrome were selected for the study according to their age, five of them were not able to understand the simple given commands and instructions so, they were excluded (Fig.,1). Thirty children with Down syndrome actually participated in the study, their age ranged from 7 to 10 years. They were selected from the Outpatient clinic, Faculty of physical therapy, Cairo university. They were able to understand the simple verbal commands, Their Body Mass Index (BMI) was in normal range (15.3 ± 0.53 and 15.68 ± 1.22 for group A and B respectively) They were able to stand momently but with history of frequent falling during walking. The children were excluded if they had anyof the following criteria: atlanto axial instability, severe ligament laxity, un controlled cardiopulmonary problems. The three stability indexes of balance and postural stability were measured for children of both group by the BBS before and after 3 successive months of conduction of the treatment program.

**Fig. 1:**
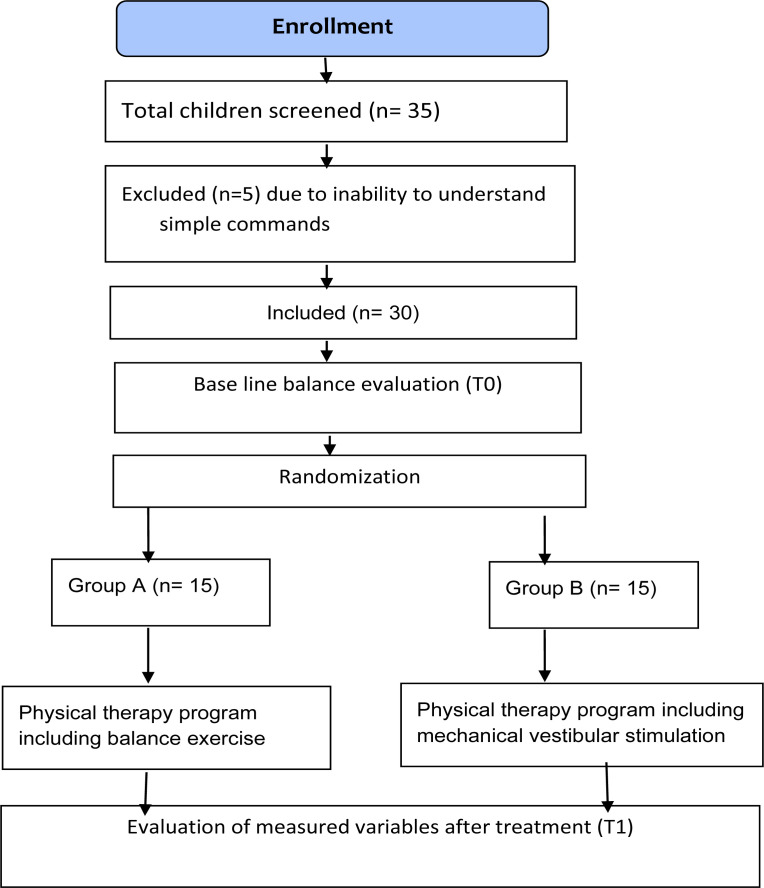
participants flow chart:

## Randomization

Children were divided randomly and equally into 2 groups (A and B). To avoid bias in group randomization, children names were introduced into the computer, then it randomly assigns them into group A and group B by means of the SPSS (Statistical Package of Social Studies) program which generated two random groups. To avoid bias, the results were analyzed by a statistical analyst who was not included in the research.

### Sample size estimation

#### Sample size

Sample size calculation was performed prior to the commencement of the actual study based on results of a pilot study on five subjects using G*POWER statistical software (version 3.1.9.2; Franz Faul, Universität Kiel, Germany) and revealed that the appropriate required sample size was thirty. The Calculations were made using α=0.05, β=0.2 and with large effect size.

### A- Measurement

#### 1- Standard weight and height scale

a valid and reliable weight and height scale was used to measure the weight and height of the children of both groups prior to the evaluation of balance.

#### 2-Biodex Balance System

Balance indices were measured using Biodex Balance System (BBS) (Biodex, Shirley, NY) which is an instrument designed to measure and train the postural stability on unstable surface. It consisted of a circular platform that is free to move in the anterior-posterior and medial lateral axes simultaneously. The device is interfaced with dedicated software (Biodex, Version 1.08, Biodex, Inc.) allowing it to measure the degree of tilt in each axis, providing an average score of postural sway. Eight springs located underneath the outer edge of the platform provide the resistance to movement (stability level of the platform). Stability levels ranged from 8 (most stable) to 1 (least stable). It has a display to give feedback about the posture sway.

### Procedure

The children were asked to stand on the platform of the device supporting on their two legs and looking forward at the display. All trials were done without shoes and feet position was recorded using coordinates on the platform's grid to ensure the same stance on future tests. In this research we used the postural stability test in which it was done with three trials of 20 seconds of each, ten seconds rest between tests and a stability level of the platform of 8 then progress to 4. In the test the platform was moving in all directions to record the three stabilityindexes (anteroposterior, mediolateral and overall). These indexes represented the amount of displacement around the of the platform that was established before testing when the platform was stable.

### For Treatment

Treatment was conducted for both groups for 1 hour/ 3 sessions per week for successive 3 months. Both groups had received 45 minutes for the traditional exercise program as follows: (strengthening exercise for abdomen and back, facilitation of postural reactions which are essential component of postural control, changing positions as rising from sitting to standing, transferring from quadruped to kneeling then to standing positions with gait training and climbing up and down stairs)^12^. Group A had received 15 minutes for regular balance exercises (standing on one limb, standing on balance board and walking on beam) in addition to the 45 minutes for the traditional exercise program. Group B had the traditional exercise program for 45 minutes in addition to 15 minutes for mechanical vestibular stimulation (forward backward, mediolateral and spinning).

### Mechanical vestibular stimulation system

It is a mechanical rotation system, in which apparatus configured to rotate around three dimensions (forward backward, side to side and spinning). The device is rotatably coupled to a frame. The vestibular stimulation devices described here are configured to allow rotation independently in two or three axes of rotation. The device is further configured to rotate continuously through 360 degrees of freedom in each axis of rotation at independently controlled accelerations for vestibular stimulation.

### Procedures of mechanical vestibular stimulation

The child was placed in sitting position on the disc swing and his hands grasping the ropes at the sides then the therapist stood behind him and begin pushing the platform in fast and jerky movement in back and front, side to side and in spinning directions with the child trying to maintain his balance in all different directions. The subject received pushing for each direction for five minutes; the total mechanical vestibular session duration was 15 minutes.

### Statistical analysis

Descriptive statistics and unpaired t-test were conducted for comparison of subject characteristics between both groups. Chi- square test was carried out for comparison of sex distribution between groups. Unpaired t-test was conducted to compare the mean values of APSI, MLSI and OASI between the control and study groups. Paired t-test was conducted for comparison between pre- and post-treatment in each group. The level of significance for all statistical tests was set at p < 0.05. All statistical analysis was conducted through the statistical package for social studies (SPSS) version 25 for windows (IBM SPSS, Chicago, IL, USA).

## Results

### Subject characteristics

Table (1) showed the general characteristics of group A and B. There was no significant difference between both groups regarding the age, weight, height and BMI (p > 0.05). Also, there was no significant difference in sex distribution between groups (p > 0.05).

**Table 1:** Basic characteristics of participants

	Group A	Group B	p-value
Age, mean± (SD), years	8.26 ± 1.16	8.33 ± 1.11	0.87
Weight, mean± (SD), kg	23.33 ± 1.34	23.6 ± 1.95	0.66
Height, mean± (SD), cm	123.7 ± 3.36	123 ± 4.35	0.64
BMI, mean± (SD), kg/m^2^	15.3 ± 0.53	15.6 ± 1.22	0.27
Sex, n (%)			
Girls	4 (30%)	5 (30%)	
Boys	11 (70%)	10 (70%)	0.23

SD, standard deviation; p-value, level of significance

### Effect of treatment on the balance stability indexes Within group comparison

**Figure 2:**
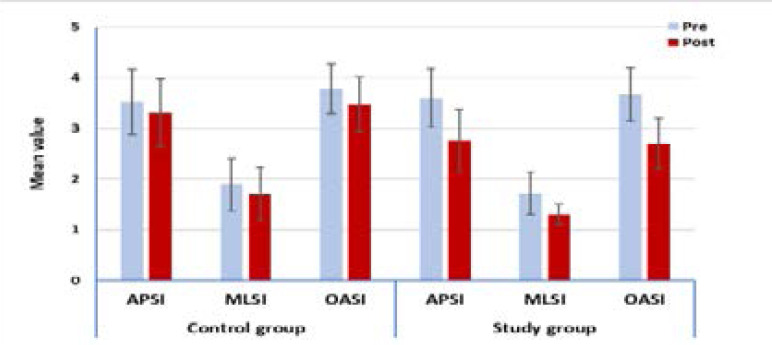
Mean APSI, MLSI and OASI pre- and post-treatment of the control and study group.

There was a significant decrease in the measured variables post treatment compared with that pretreatment results in both groups (p > 0.0001). The percent of decrease in anteroposterior stability index (APSI), mediolateral stability index (MLSI) and overall stability index (OASI) in the control group were 6.25,10.05and 7.94% respectively, while that in the study group were 23.61, 24.42and 26.43% respectively (Table 2) Fig.2.

**Table 2:** Mean APSI, MLSI and OASI pre- and post-treatment of both groups

	Group A	Group B			
				
	± SD	± SD	MD	t- value	p value
**APSI**					
**Pre treatment**	3.52 ± 0.65	3.6 ± 0.58	-0.08	-0.32	0.74
**Post treatment**	3.3 ± 0.67	2.75 ± 0.62	0.55	2.33	0.02
**MD**	0.22	0.85			
**% of change**	6.25%	23.61%			
**t- value**	7.05	12.54			
	***p = 0.0001***	***p = 0.0001***			
**MLSI**					
**Pre treatment**	1.89 ± 0.52	1.72 ± 0.41	0.17	0.96	0.34
**Post treatment**	1.7 ± 0.53	1.3 ± 0.2	0.4	2.72	0.01
**MD**	0.19	0.42			
**% of change**	10.05%	24.42%			
**t- value**	5.39	5.84			
	***p = 0.0001***	***p = 0.0001***			
**OASI**					
**Pre treatment**	3.78 ± 0.49	3.67 ± 0.52	0.11	0.6	0.54
**Post treatment**	3.48 ± 0.54	2.7 ± 0.5	0.78	4.19	0.0001
**MD**	0.3	0.97			
**% of change**	7.94%	26.43%			
**t- value**	8.52	8.19			
	***p = 0.0001***	***p = 0.001***			

mean; SD, standard deviation; MD, mean difference; p-value, probability value

### Between groups comparison

There was no significant difference in the three stability indexes between both groups pre-treatment (p > 0.05). Comparison between both groups post treatment revealed a significant decrease in all measured variables of group B whencompared with that of group A (p > 0.05) (Table 2).

The present study attempted to identify the benefits of using mechanical vestibular stimulation over the traditional balance exercises on improving postural stability and balance when added to the treatment program for children with Down syndrome, study included thirty children with Down syndrome with age ranged from 7 to 10 years old, Group A had received the traditional exercise program with regular balance exercises while group B has the same exercise program but with mechanical vestibular stimulation instead of the regular balance exercises. Evaluation of the postural stability indexes was done by BBS before and after treatment.

The pre-treatment findings of the measured indexes for the present study were matching with those reported by Rose et al.^15^, who reported that high stability indices on the Biodex balance system indicate a lot of movements during the test (increase of sway in different directions) and therefore less postural stability.

The significant improvement in the post treatment measured variables for both groups indicated that the physical therapy exercise program including the balance exercises is effective in enhancing the postural stability in children with Down syndrome by increasing their abilities to regain body position when exposed to balance disturbance, This agrees with Lord et al. 16, who observed that the balance training program is one of the intrinsic parts of motor skills acquisition because it facilitates the normal weight shift, mobility and provide the balance mechanisms when center of gravity is disturbed. Enhancing the abilities of those children to control their balance is important because better balance will decrease their fear of falling or getting hurt and thereby increasing their desire to participate in physical activities.

Balance and stability are often a major problem for children with Down syndrome due to the characteristics associated with this syndrome such as low muscle tone, poor balance, perceptual difficulties with inadequate vision and hearing^18^. Therefore, physical therapy intervention programs should include different exercises to improve balance for those children aiming at improving their functional abilities to reach a physically active life. Balance was selected to be evaluated in this study because of its important role in all motor functions. this is supported by Westcott et al.^19^, who reported that balance is an integral part of all motor abilities as any defect in balance will be reflected on all activities of daily living,

Improvement in post treatment mean values of group B may be attributed to the effect of spinning in enhancing trunk proprioception and increase the activity of antigravity muscles which counteract the force of gravity and leads to modulation of postural tone. That agrees with (Panichi et al.^20^, who stated that the perception of body rotation is enhanced by proprioceptive input due to muscles' action (turning trunk, to each side) rather than of their static anatomical position. This effect increasing the gain of the perception of motion in the presence of intense active rotation of the body, when the body movement must follow the direction in which the head turns that may require a superior perception for a better performance of the desired movement. It can be suggested that integration of mechanical vestibular stimulation program with the designed physical therapy program has a significant effect on balance in children with Down syndrome, so it should be considered as an important therapeutic modality for treatment of these cases and adding mechanical vestibular stimulation to the treatment sessions of Down syndrome is highly recommended.

Recently, studies have shown differences in balance control through internal and external attentional strategies. Instructions that direct attention to body movements (internal focus), such as giving instructions to learners in relation to their body movements has not been shown to be the most effective way of learning as those directing the learner's attention to the movement effects (external focus). However, when instructions direct the learner's attention to the effects of their movements on the environment has been found to cause more effective learning^21^. Using the mechanical stimulation of the vestibular system helped the child to better integrate the different sensory stimuli that he received from the surrounding environment, as increase of the vestibular stimulation causes the enhancement of the arousal and attention of the child to balance perturbance so he can modulate his position to guard against falling.

Oscillations caused by mechanical vestibular stimulation when done irregular and fast and arrhythmic help in facilitation of muscle tone which reflected in improve postural control and stability that was recorded by decrease degree of center of gravity deviations in forward backward and mediolateral directions after treatment that agrees with Herdman^22^, who stated that the vestibular is connected with auditory, visual, proprioceptive and motor system. The vestibular system has been found affecting muscle tone, visual gaze and body head spatial orientation in space.

Forward, backward and rotational movement used in the mechanical vestibular stimulation program for group B was effective in improving the organization of sensory information from vision, proprioceptive as well as the vestibular that helped in controlling balance which agreed with^23^. Vestibular stimulation could be achieved through both linear, angular motion or through change of head position. Vibration also causes vestibular stimulation. So, mechanical vestibular stimulation is used in physical therapy for enhancing the development of postural control and balance.

### Implication on practice

Balance is representing a major problem for children with Down syndrome. Therefore, the interventions used for them should provide a direction for improving this needed physical function to enable active life participation.

## Conclusion

Adding mechanical vestibular stimulation to the treatment program for children with Down syndrome is beneficial for improvement of their balance and postural stability.

## Limitations

This study was limited by the lack of a functional scale to evaluate the balance in children with Down syndrome.
